# Outcomes of Renal Transplantation in Brunei Darussalam over a Twenty-Year Period (1993–2012)

**DOI:** 10.1155/2014/784805

**Published:** 2014-11-12

**Authors:** Jackson Tan, Muhammad Abdul Mabood Khalil, Si Yen Tan, Muhammad Khalil, Dalinatul Ahmed, Shaukat Zinna, William Chong

**Affiliations:** ^1^RIPAS Hospital, Bandar Seri Begawan BA1710, Brunei Darussalam; ^2^Aga Khan University Hospital, Karachi 74800, Pakistan; ^3^Prince Court Medical Centre, 50450 Kuala Lumpur, Malaysia

## Abstract

*Objectives*. Brunei Darussalam has a high prevalence and incidence of end stage renal disease (ESRD). Up until 2012, all renal transplantations were performed in overseas centres, either as government-sponsored (living-related transplantation) or as self-sponsored (commercialized transplantation) ones. We hypothesize that graft and patient survival of Brunei renal transplant patients are on a par with international standards.* Materials and Methods*. Data of all renal transplant patients in Brunei were analysed over a twenty-year period from registry records and case notes. Comparative survival data from other countries were obtained from PubMed-listed literature.* Results*. A total of 49 transplantation procedures were performed in foreign centres between 1993 and 2012. 29 were government-sponsored and 20 were self-sponsored transplantations. The 5- and 10-year overall patient survival rates were 93.3% and 90.1%, respectively. The 5- and 10-year overall graft survival rates were 91.1% and 81.2%. There is no difference in the survival outcomes of government-sponsored and self-sponsored patients. Living-related (government-sponsored) and commercialised (self-sponsored) grafts had equivalent survival to those reported in the literature.* Conclusion*. Our survival data was on par with those achieved in many countries. We hope to use this information to convince local stakeholders and patients to favour transplantation as the preferred modality of RRT.

## 1. Background

Brunei Darussalam has a high prevalence and incidence of end stage renal disease (ESRD). Data from the 1st Brunei Dialysis and Transplant Registry (BDTR) revealed a prevalence and incidence of 1250 and 265 per million population (pmp), respectively, in 2011 [1]. Renal transplantation is a poorly utilised and accepted modality of renal replacement therapy (RRT) in Brunei. At the end of 2012, only 6% of the existing RRT population had a functioning transplant graft [2]. The discrepancy between the high prevalence of ESRD and low prevalence of renal transplantation has led to overdependence on haemodialysis (HD) and peritoneal dialysis (PD) therapy. Eligible ESRD patients desirous of having a renal transplant, and their screened and matched relatives, are sent under government sponsorship to endorsed overseas centres for living-related transplant operations. This practice has been increasingly prevalent in the last twenty years, especially with the growing pool of eligible ESRD patients, improving transplant awareness and the absence of a local transplant program. In addition, there are a substantial number of noneligible patients (usually those without suitable kidney donors) who go overseas for commercialised and non-government-sponsored transplantations.

A local living-related transplant program was started in Brunei in 2013 [3]. The main motivations behind starting the program are to provide care for patients in their local environment and to deter commercialised overseas transplantations. At present, there is no cadaveric transplant program in Brunei. As the program is still at its infancy, there is a limit to the number of transplants that can be performed due to the lack of resources, expertise, and facilities. Patients are still being sent abroad as demand is outstripping the capability of the local transplant centre. While waiting for the local program to mature, the country will continue to be reliant on government-sponsored overseas transplant in the immediate future. In doing so, extra justifications to support the cost-effectiveness and safety of this practice are needed, which has provided the impetus for this study to be undertaken.

We hypothesize that graft and patient survival of Brunei transplant patients performed overseas are on a par with international acceptable standards. The research also enables us to review our renal transplantation practice and trend in Brunei Darussalam over the last twenty years (1993 to 2012).

## 2. Methods and Objectives

We analysed data of all renal transplant patients in Brunei over a twenty-year period (between 1993 and 2012). Data collection was performed through the combination of the use of registry data, patients' case notes, and old department records. The data collected included age at transplant, gender, aetiology of primary renal disease, country of transplant, date of transplant, date of death (if applicable), and date of graft failure. For the ease of comparisons, we divided the patients into two groups: government-sponsored patients and self-sponsored (commercialised) patients. Comparative data from other countries were obtained from the PubMed-listed literature. Statistical analysis on survival outcomes was performed using Kaplan-Meier survival analysis on PASW Statistics 18 software. Fisher's test was used for statistical analysis of demographic data.

## 3. Results

A total of 49 transplantation procedures were performed on 47 patients between 1993 and 2012. All the procedures were performed in foreign centres. There was male preponderance (65%), especially in the self-sponsored population. The most common aetiological diseases for transplant patients were glomerulonephritis (47%), diabetes mellitus (6%), and hypertension (4%). All self-sponsored patients received kidneys from unrelated sources. Chinese patients were more likely to have self-sponsored transplantation (*P* < 0.05) than government-sponsored transplantation. Most of our transplanted patients (49%) had their surgeries performed in Singapore. The demographic details of these patients are illustrated in Table 1. Transplantations performed in the earlier part of the researched period (1993–2000) were mainly self-sponsored. However, there appeared to be a trend towards government-sponsored transplantation in the last decade. The incidence of government-sponsored and self-sponsored transplantations between 1993 and 2012 is summarised in Table 2.

**Figure 1:**
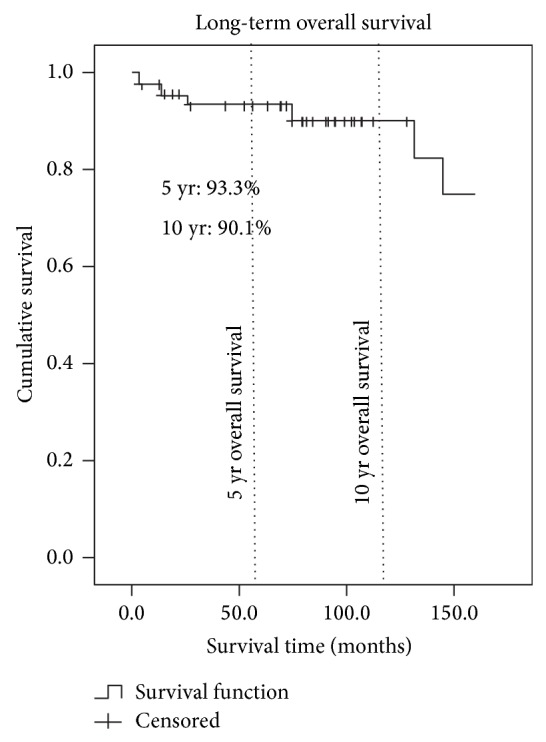
Kaplan-Meier overall patient survival curve of patients after renal transplantation.

**Figure 2:**
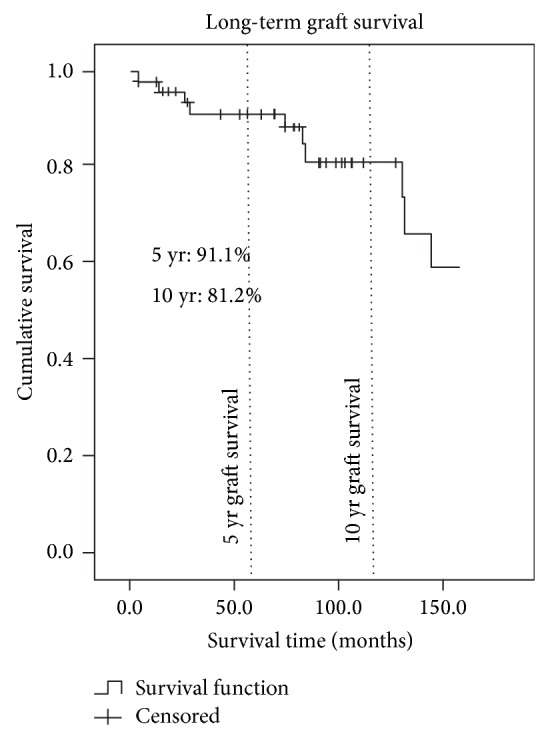
Kaplan-Meier graft survival curve of patients after renal transplantation.

**Figure 3:**
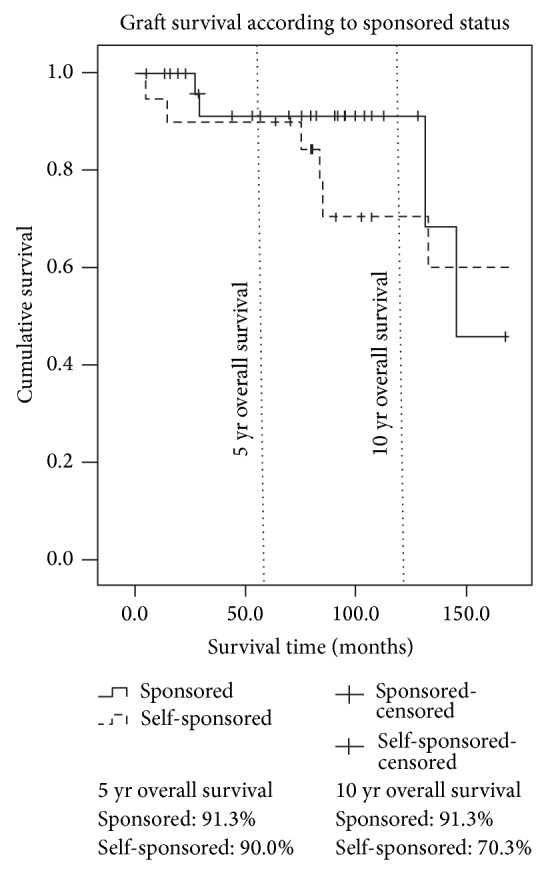
Kaplan-Meier graft survival curves for government-sponsored and self-sponsored transplantations.

The 5- and 10-year patient survival rates were 93.3% and 90.1%, respectively (Figure 1). The 5- and 10-year graft survival rates were 91.1% and 81.2% (Figure 2). The 5- and 10-year graft survival rates for government-sponsored patients were 91.3% and 91.3%, respectively. Most of the failed grafts for government-sponsored patients were within the first three years of transplant and beyond ten years. The 5- and 10-year graft survival for self-sponsored patients was 90.0% and 70.3%, respectively. There are, however, no statistical differences between the two groups.  Figure 3 highlighted the difference between these two groups. The 5-year graft survival for genetically related, spousal, and unrelated transplantations (commercialised) was 92.9%, 87.5%, and 90.0%, respectively. There was no statistical difference between these groups.

The 5-year living-related (government-sponsored) patient and graft survival were equivalent to data from Singapore, Malaysia, USA, UK, Australia, and New Zealand. Our 10-year graft and patient survival were superior (Table 3). The 5- and 10-year commercialised (self-sponsored) patient and graft survival were also equivalent to Singapore and Malaysia but superior to other countries (Saudi Arabia, Iran, Republic of Korea, Turkey, and Pakistan (Table 4)).

## 4. Discussion

Government-sponsored transplant patients were patients that had met the fitness and citizenship eligibility criteria and had a willing donor from their immediate family. These patients were usually sent to Singapore or Malaysia. Self-sponsored patients were those who did not fulfil these criteria and partook in commercialised transplantations. In these instances, there was usually a lack of communication between the foreign transplant centres and the patients tending to be evasive in giving details of the donors (either deceased or living-unrelated). For the purpose of this discussion and easy comparisons with international literature, we will classify government-sponsored patients as living-related transplantations (LD) (Table 3) and nonsponsored patients as commercialised transplantations (Table 4).

Our 5- and 10-year overall graft survival rates were 91.1% and 81.2%, respectively. The 5- and 10-year overall patient survivals were 93.3% and 90.1%, respectively. We believe that these rates were respectable when compared to those achieved in many countries. Our 5-year living donor (LD) graft and patient survival rates were equivalent to those achieved in Singapore [4], where the majority of our government-sponsored patients (93.3%) were sent. Our 10-year LD graft and patient survival rates were better than those reported by the Malaysian Dialysis and Transplant Registry (MDTR) [5], Organ Procurement and Transplantation Network (OPTN) [6], UK Renal Registry [7], and ANZ Renal Registry [8]. This could be explained by the greater proportion of younger (median age 32 years) and nondiabetic patients (96.6%) in our LD transplant population. Patients who are deemed to be with high risk or have complicated medical issues are not sent for transplantation overseas. This means that the average Brunei LD transplant patient is comparatively younger and healthier than the average international transplant patient, which could explain the superior survival rates. Table 3 shows the LD transplantation survival rates of different countries.

Our data did not show any significant survival differences between government-sponsored and self-sponsored (or commercialised) groups. This may indicate that a significant proportion of the kidneys from commercialised transplantation may have been derived from living rather than deceased donors (DD). Similar survival trends were also observed in neighbouring Southeast Asian countries (Singapore and Malaysia) with a significant commercialised kidney transplant population. Vathsala [9] reported a slightly superior survival in the DD transplantations from China when compared to locally performed DD transplantations in Singapore. However this advantage disappeared when DD transplanted patients with primary nonfunction or vascular thrombosis were excluded from the analysis. Morad and Lim [10] reported equivalent survival rates between commercialised (*n* = 389) and noncommercialised patients in Malaysia with no difference in infection rates. More recent data from the MDTR [5] showed a similar trend with a 5-year survival rate of 87% in commercialised kidney transplants. Similar results also stemmed from studies from Saudi Arabia [11], Iran [12, 13], and Republic of Korea [14]. However, studies from Turkey [15, 16] and Pakistan [17] showed inferior rates of patient and graft survival with high rates of complications. Table 4 summarises the graft and patient survival between commercialised and noncommercialised transplants in studies with >100 patients in the last twenty years.

The lack of survival differences between the two groups could be explained by several factors. We may not have an accurate representation of early failed commercialised transplants (primary graft failure or venous thrombosis) due to poor or lack of communication with these centres. We also speculate that wealthier, motivated, and educated patients are more likely to seek overseas commercialised transplantations. These patients can usually afford a healthier lifestyle and have access to better private healthcare facilities. They are less likely to spend a long time on dialysis and will have avoided the usual dialysis-related atherosclerotic complications that may jeopardise long-term kidney and patient survival. We believe that these factors amongst the self-sponsored patients may have led to misleadingly good outcomes when compared with self-sponsored LD transplants that theoretically should have undergone more stringent cross-matching exercises and had less HLA mismatch.

It is important for us to demonstrate to healthcare providers and patients in the country that renal transplantation is superior to the other modalities of RRT. There has been an undersubscription of renal transplantation as the preferred RRT modality over the past few decades. Between 2008 and 2012, the country's prevalent RRT population increased by 24% from 502 to 620 patients. During the same time period, the prevalent renal transplant population increased by only 13% from 31 to 35 patients [2]. There was a fall in the number of transplants since 2009 which could be related to the difficulties in procuring commercial kidneys in India and China. This could also be through our persistent efforts in educating patients to deter commercialised transplant activities. This is consistent with trends observed in other countries, likely as a result of international efforts to curb illegal transplantations [18]. Our current transplant rate of 9 ppm in 2011 (based on a Brunei population of 422,700 [19]) was low compared to other developed countries like USA (57.5), UK (53.3), and Australia (37.9) [20]. However, our rates were similar to those achieved by other neighbouring Southeast Asian countries like Malaysia (8.3), Singapore (18.5), and Thailand (5.5) [20]. We recognise that this trend is in part due to poor public awareness, inadequate cultural acceptance, and the lack of local transplant facilities.

Considering the benefits of renal transplantation, numerous public exercises have been carried out to improve public awareness. A recent public survey succeeded in disseminating information about renal transplantation to the general public [21]. The same survey revealed that 78.7% of 300 respondents were willing to donate their kidneys if the need arose and 59.7% of respondents preferred to have transplantation locally. We also conducted a quality of life survey amongst RRT patients which showed that transplant patients had a superior quality of life when compared to HD and PD patients in Brunei [22]. This piece of information has been disseminated in public forums and health promotion events to encourage patients and relatives to appreciate the benefits of transplantation. We also intend to use the results of this study to convince the public that equivalent international survival outcomes can be achieved through the practice of legitimate overseas transplant and that the local expertise in the country is capable of monitoring and treating long-term transplant patients. The encouraging results from our recent studies have given extra motivation and drive to local service providers to proliferate and expand the local transplant program to enhance the quality of care given to local ESRD patients.

## 5. Conclusion

Our survival analysis showed noninferior rates compared to those achieved in other countries in both our LD and commercialised transplant populations. We hope to use this information to convince local stakeholders to invest more interest and resources to favour transplantation as the preferred modality of RRT. The favourable transplant outcomes may also help to enhance public awareness and galvanise patients' interest in choosing transplantation over the other modalities of RRT. This study has also provided baseline survival data to enable future comparisons with our fledging local program.

## Figures and Tables

**Table 1:** Demographic details of transplant patients.

	Total	Government-sponsored	Self-sponsored	*P* value
Number	49	29	20	
Median age (years)	31	32	37	
Sex				
Male	32	16	16	>0.05
Female	17	13	4	
Relationship to recipient				
Spouse	9	9	0	<0.001
Relatives	20	20	0	
Unknown	20	0	20	
Aetiology				
Diabetes mellitus	3	1	2	*P* > 0.05, for trend
Hypertension	2	1	1	
Glomerulonephritis	23	15	8	
Others	3	2	1	
Unknown	18	10	8	
Race				
Malay	37	27	10	0.014
Chinese	12	2	10	
Place where transplant was performed				
Singapore	29	28	1	*P* > 0.05, for trend
Malaysia	2	2	0	
China	9	0	9	
India	7	0	7	
Others	2	0	2	

**Table 2:** Incident cases of government-sponsored and self-sponsored transplantations between 1993 and 2012.

Time period	Government-sponsored patients	Self-sponsored patients	Total patients
1993–1996	2	5	7
1997–2000	2	4	6
2001–2004	4	4	8
2005–2008	12	6	18
2009–2012	9	1	10

All	29	20	49

**Table 3:** Living donor (LD) graft and patient survivals.

Country	Year	Source	Year of transplant	5-year graft survival	5-year patient survival	Year of transplant	10-year graft survival	10-year patient survival
Brunei	2013	Tan [1]	1993–2012	91.3	95.7	1993–2012	91.3	95.7
Singapore	2009	Vathsala and Khuan [4]	1999–2006	95.3	96.6	NA	NA	NA
Malaysia	2013	MDTR [5]	1993–2012	86	94	1993–2012	71	88
USA	2012	Organ Procurement and Transplantation Network [6]	2006	84	93.4	2001	60.3	83
UK	2012	UK Renal Registry [7]	2002–2006	91	96	NA	NA	NA
Australia and New Zealand	2012	Australia and New Zealand Dialysis and Transplant Registry [8]	2000–2004	87.7	94.3	2000–2004	72.4	87.2

**Table 4:** Commercialised graft and patient survivals.

Authors	Year	Country	Journal	Number	Country of transplant	Graft outcome	Patient outcome
Tan [1]	2013	Brunei		20	China, India, Indonesia, Philippines	5- and 10-year graft survival at 90% and 70.3%	5- and 10-year patient survival at 94.5% and 83.5%
Vathsala [9]	2010	Singapore	Clin Transpl	192	China, India	5- and 10-year graft survivals for DD transplants from China	NA
Morad and Lim [10]	2000	Malaysia	Transplant Proc	389	Not discussed	5-year survival at 72%	5-year survival at 82%
MDTR [5]	2012	Malaysia	MDTR		Not discussed	5-year graft survival at 87%	NA
Quinibi [11]	1997	Saudi Arabia	Clin Transpl	540	India	5-year survival at 72%	5-year survival at 92%
Ghods and Savaj [12]	2006	Iran	Clin J Am Soc Nephrol	1499	Iran	Graft 5- and 10-year survival at 74.4% and 48.8%, respectively	Patient 5- and 10-year survival at 87.1% and 72.2%
Ghods [13]	2002	Iran	Nephrol Dial Transplant	942	Iran	5- and 10-year survival at 64.2% and 43.7%, respectively	5- and 10-year survival at 83.7% and 73.3%, respectively
Kwon et al. [14]	2011	Republic of Korea	J Korean Med Sci	462	China	Survival of 96.5% (median follow-up 21.2 months)	Survival of 96.8% (median follow-up 21.2 months)
Sever et al. [15]	2001	Turkey	Kidney Int	115	India, Iraq, Iran	2-, 5-, and 7-year survival at 84%, 66%, and 53%, respectively	2-, 5-, and 7-year survival at 90%, 80%, and 74%, respectively
Çolako$\overset{ˇ}{\text{g}}$lu et al. [16]	1998	Turkey	Nephron	127	India	5-year survival at 57%	5-year survival at 92%
Rizvi et al. [17]	2009	Pakistan	Transpl Int	126	Pakistan	Graft 1- and 5-year survival at 86% and 45%, respectively	NA
